# The PROTEGE program, A Gatekeeper Program for Suicide Prevention: Validity evidence from Focus Group Evidence

**DOI:** 10.1192/j.eurpsy.2026.10691

**Published:** 2026-07-31

**Authors:** G. V. R. Toro, L. Gonzalez-Agudelo, P. Arias, N. Lagunas, T. Vieites, A. R. Traistaru-Descultu, A. De La Torre-Luque

**Affiliations:** Complutense University of Madrid, Madrid, Spain

## Abstract

**Introduction:**

PROTEGE constitutes a brief gatekeeper program on suicide prevention literacy and skills, intended to train secondary school teachers to identify, assess, and appropriately initiate referral paths for adolescents at risk of suicidal behavior. The program is 6-hour long and combines theoretical and practical contents, including real-case discussions and role-play activities.

**Objectives:**

This study aims to provide qualitative evidence on the usefulness and feasibility of the PROTEGE program from relevant stakeholders in school-based settings.

**Methods:**

PROTEGE was evaluated on a 2-hour focus group, with 11 experts and stakeholders that included clinical psychologists, educational psychologists, teachers, school administrators, a psychiatrist, and a bereaved parent who had lost a child due to suicide. Participants received the training materials and were asked to review and evaluate the program using in terms of clarity and comprehension, relevance and applicability, methodology and materials, cultural adaptation, and overall satisfaction. In addition, participants rated the program’s potential to prepare educators to identify and support at-risk students and indicated whether they would recommend it. An open-ended question invited further suggestions for improvement.

**Results:**

The PROTEGE program was evaluated very positively across most items. For clarity, 63.6% strongly agreed that the content was easy to understand, while 72.7% agreed that the information was sufficient, and 72.7% strongly agreed that the key concepts were accessible. Regarding relevance, 18.2% agreed and 81.8% strongly agreed the program was pertinent to their context, while 54.5% agreed and 36.4% strongly agreed that the strategies were applicable. Methodology and materials were well valued, with 91% endorsement of the program structure. Cultural adaptation received somewhat mixed views, with most agreeing (72.7%) or strongly agreeing (9.1%) that the program reflected the local context, while over half (54.5%) strongly agreed and 27.3% agreed that the program would benefit from cultural or contextual improvements. Finally, 72.7% agreed and 27.3% strongly agreed that the program met expectations. All participants stated they would recommend the program to other professionals.

**Conclusions:**

Overall, the PROTEGE program was evaluated positively by relevant experts and stakeholders. The combination of theoretical instruction and interactive methods, such as case discussions and role-playing, was considered effective to enhance teachers’ ability to identify and support students at risk of suicide. Although some participants suggested improvements related to cultural adaptation, the realism of activities, and the presentation format, overall satisfaction was very high. These findings suggest that PROTEGE is a feasible and well-received initiative with strong potential to strengthen suicide prevention efforts in schools.

**Disclosure of Interest:**

None Declared

